# Predicting Low Birth Weight in Big Cities in the United States Using a Machine Learning Approach

**DOI:** 10.3390/ijerph22060934

**Published:** 2025-06-13

**Authors:** Yulia Treister-Goltzman

**Affiliations:** 1 Department of Family Medicine and Siaal Research Center for Family Practice and Primary Care, The Haim Doron Division of Community Health, Faculty of Health Sciences, Ben-Gurion University of the Negev, Beer-Sheva 84161, Israel; yuliatr@walla.com; Tel.: +972-8-6477436; Fax: +972-8-6477636; 2 Clalit Health Services, Southern District, Beer-Sheva 84161, Israel

**Keywords:** low birth weight, big cities, USA, machine learning, prediction, influential predictors

## Abstract

Objective: Low birth weight is a serious public health problem even in developed countries. The objective of this study was to assess the ability of machine learning to predict low birth weight rates in big cities in the USA on an ecological/population level. Study design: The study was based on publicly available data from the Big Cities Health Inventory Data Platform. The collected data related to the 35 largest, most urban cities in the United States from 2010 to 2022. The model-agnostic approach was used to assess and visualize the magnitude and direction of the most influential predictors. Results: The models showed excellent performance with R-squared values of 0.82, 0.81, 0.81, and 0.79, and residual root mean squared error values of 1.06, 0.87, 1.03, 0.99 for KNN, Best subset, Lasso, and XGBoost, respectively. It is noteworthy that the Best subset selection approach had a high RSq and the lowest residual root mean squared error, with only a four-predictor subset. Influential predictors that appeared in three/four models were rate of chlamydia infection, racial segregation, prenatal care, percentage of single-parent families, and poverty. Other important predictors were the rate of violent crimes, life expectancy, mental distress, income inequality, hazardous air quality, prevalence of hypertension, percent of foreign-born citizens, and smoking. This study was limited by the unavailability of data on gestational age. Conclusions: The machine learning algorithms showed excellent performance for the prediction of low birth weight rate in big cities. The identification of influential predictors can help local and state authorities and health policy decision makers to more effectively tackle this important health problem.

## 1. Introduction

Low birth weight (LBW), defined as babies with a birth weight under 2500 g, is a well-acknowledged risk-factor for perinatal and infant mortality, and for subsequent cardio-vascular, respiratory and cognitive disorders in adulthood. In 2020, 19.8 million newborns, an estimated 14.7% of all babies born worldwide, had LBW. Global progress on reducing LBW prevalence has been slow. In some regions, including South Asia, West and Central Africa, Eastern and Southern Africa, Europe, and Central Asia, the prevalence of LBW decreased slightly from 2000 to 2020. Several other regions, including Latin America, the Caribbean, North America, the Middle East, North Africa, East Asia and the Pacific demonstrated no change or a slight increase in prevalence from 2000 to 2020 [1]. In the United States the prevalence of LBW increased between 2016 and 2022 from 7.6% to 8.2% [2]. Preterm birth and LBW remained the second leading cause of infant death in the United States, accounting for 15% of infant deaths in 2021. The infant mortality rate among infants with LBW in the United States was 41.8 per 1000 live births in 2021, even higher among short-gestation and LBW infants (80.7 per 1000 live births) [3]. A large cohort study that assessed long-term morbidity associated with LBW showed significantly increased risks for obesity, hypertriglyceridemia, high LDL-cholesterol, high blood pressure, metabolic syndrome, non-alcoholic fatty liver disease, allergic and atopic symptoms, and lack of tertiary education, with risks increased by 1.11–1.35 for different pathologies [4]. LBW is a multifactorial phenomenon and many maternal, environmental, and social factors have been linked to LBW. The main maternal factors associated with LBW are malnutrition/poor diet, parity, obstetric history and antenatal care, infectious diseases, including sexually transmitted diseases, smoking, alcohol abuse, maternal age, and mental distress [5,6,7]. Hypertension during pregnancy is one of the leading factors for LBW, while diabetes mellitus and obesity are usually associated with higher infant birth weight [8,9]. Environmental factors such as ambient air pollution and extreme climate conditions can also have an impact [10]. Among social factors, low education, low income, and low socio-economic status were reported to be related to increased risk of LBW [7,11]. As a serious public health problem, even in developed countries [12], LBW stands at the intersection between medicine, environmental sciences, and sociology, hence a better understanding of this problem on the population level is crucial. The machine learning (ML) approach is a very promising tool to deal with complex and high-dimensional data and has already been introduced to predict LBW in several studies [11,13,14,15] in which multiple ML approaches were tested. These studies were carried out on an individual, not a population, level. They evaluated the performance of classifier algorithms, as LBW was treated as a binary outcome. The XGBoost algorithm showed an excellent predictive performance in two studies [11,14], as did the random forest in two studies [13,14], the deep learning feed forward in one study [13], and the category boosting and gradient boosting decision tree in one study [15]. In contrast, the logistic regression and decision tree showed average performance metrics [11,13,14,15].

Ecological studies, in which the unit of observation is the population or the community, allow for the comparison of aggregated data across different areas and the investigation of population-level exposures. Predicting LBW at a population level can direct and focus interdisciplinary initiatives at state and local levels, enabling timely interventions and mitigating adverse outcomes.

The primary goal of the present study was to assess the performance of ML approaches in predicting LBW in big US cities at a population level and to compare the predictive ability of different models. The secondary goal was to assess the relative importance of the contributing factors, highlighting the most important variables in the prediction process.

## 2. Methods

This ecological study was based on publicly available data from the Big Cities Health Inventory Data Platform (BCHI) [16]. The BCHI was launched by the Big Cities Health Coalition with funding from the Centers for Disease Control and Prevention and is now maintained by the Drexel University Urban Health Collaborative in partnership with the Big Cities Health Coalition. BCHI contains over 150,000 standardized data points for more than 120 health social, environmental, and health indices for the 35 largest, most urban cities in the United States from 2010 to 2022 [16]. Data on the rate of LBW in the BCHI are based on natality files from the National Vital Statistics System of the National Center for Health Statistics [16] and are expressed as a continuous variable of the number of LBW infants per 100 live births. Common sense, expert opinion, and findings from prior research in the domain were used to select candidate LBW predictors for this study.

Statistical analyses were carried out using R version 4.3.1, using the packages “caret”, “rsample”, “recipes”, “yardstick”, “randomForest”, “xgboost”, “leaps”, and “shapviz”.

Five statistical regression learning models—K-nearest neighbors (KNN), Best subset selection, Lasso regression, Random forest, and Extreme gradient boosting (XGBoost)—were trained. KNN is a simple and intuitive algorithm that makes predictions by finding the K nearest data points to a given input and averaging their target values [17]. Best subset selection performs selection of the best set of predictors by identifying the best model that contains a given number of predictors, where the best is quantified using the residual sum of squares [17]. Lasso stands for Least Absolute Shrinkage and Selection Operator. It facilitates automatic feature selection by adding a penalty term to the residual sum of squares, which is multiplied by the regularization parameter (lambda or λ). This shrinks some of the coefficients towards zero, thus reducing the importance or eliminating some of the features from the model altogether, resulting in automatic feature selection [17]. The bootstrapping Random Forest algorithm combines ensemble learning methods with the decision tree framework to create multiple randomly drawn decision trees from the data, averaging the results to output a new result that often leads to strong predictions [17]. Finally, XGBoost is an ensemble learning method that uses parallel gradient tree boosting and combines the predictions of multiple weak models to produce a stronger prediction [17].

The cases were randomly assigned to either the “training set” (70%) or the “test set” (30%) in all models. In the training set, internal validation was carried out with the help of 5-fold cross-validation. Several preprocessing procedures were performed. Given the dependency between observations related to the same city, data leakage of the observations from the same city was prevented between training and validation sets, during internal (cross-validation) and external (training and test set) validation. Standardization of the independent variables was carried out using the KNN and Lasso regression models, preventing features with larger magnitudes from disproportionately influencing the distance calculation in KNN, and improving the model’s ability to select important features and reducing the magnitude of the model coefficients in Lasso. The grid search method, which involves training a model for possible combinations of hyperparameters in a predefined set, was used for hyperparameter tuning.

Thirty-three potential predictors were assessed to predict the percent of infants born with LBW in the 35 big cities over 13 years (i.e., 455 observations).

Table S1 presents the description of the chosen predictors, that were grouped by four domains related to health/morbidity, climate/built environment, social/economic, and demographic factors. Missing datapoints were imputed by the value from the same variable for the same city from the nearest adjacent year.

The performance parameters of the prediction models were externally validated using the test set. Metrics, including R-squared (RSq) (the proportion of the variance of the dependent variable that can be explained by the regression), and residual root mean squared error (RMSE) (average difference between predicted and actual empirical values of data) were used to assess the predictive power of the models.

Ultimately, the best performing models were analyzed for the importance of their features. The model-agnostic approach was used. Model-agnostic methods do not account for the structure of the model, can be applied to any ML algorithms, and work on the ‘black box’ model approach. They obtain explanations by perturbing and mutating the input data and obtaining the sensitivity of the performance of these mutations with respect to the original data performance. SHAP (SHapley Additive exPlanations) values are a way to explain the output of any ML model. It uses a game theoretic approach that measures each player’s contribution to the outcome. In ML, each feature is assigned a SHAP value representing its contribution to the model’s output. Features with positive SHAP values positively impact the prediction, while those with negative values have a negative impact. The magnitude is a measure of how strong the effect is.

Many of the potential predictors could be highly correlated, i.e., obesity, hypertension, inactivity, and diabetes, or poverty and crime. Multicollinearity does not alter the predictive ability of the models, so potential multicollinearity did not affect the primary goal of the present study, i.e., assessing the performance of ML approaches in predicting LBW. Variable selection methods and modified estimator methods by regularization and penalty mechanisms are intrinsically used to solve multicollinearity in machine learning models [18,19]. It is well-known that the values of standard regression coefficients depend heavily on the collinearity of the predictors. But SHAP values, used in a model-agnostic interpretation, calculate feature contributions by averaging across all permutations of the features joining the model. This enables SHAP values to control for variable interactions. SHAP values are additive, meaning that you can attribute a portion of the model’s predictive value to each of the observation’s input variables. For example, if you have a model that is built with three input variables, then you can write the predicted value as the summation of the corresponding SHAP values plus the average predicted value across the input data set. Therefore, the secondary goal of the study, assessing the relative importance of the contributing factors, was also invulnerable to possible multicollinearity [20].

## 3. Results

Details on the missing values of the predictor variables are provided in Table S2.

Most variables had no missing values, while a few had less than 5% missing values. The only valuable with a substantial number of missing values was the uninsured population (105 (23.08%)).

Descriptive statistics of the characteristics of the study sample are presented in Table 1. The mean (SD) percent of LBW births was 9.11 (1.89), with the highest percent in Detroit ((14.2 (0.83)) and the lowest in Seattle ((6.51 (0.27)). The highest prevalence of noncommunicable diseases, such as obesity, diabetes, hypertension, and mental distress, was observed in Detroit, with means (SDs) of 35.5 (1.53), 18.00 (0.33), 46.30 (0.79), and 18.6 (1.52), respectively. Eighteen percent of the adult population in the big cities in the USA smoked and more than 25% did not engage in physical activity. Among individual cities these numbers were highest for Detroit at 29.00 (68.80) and 36.60 (1.22), respectively. Various social indices such as poverty level, unemployment, single-parent families, and violent crimes were also highest in Detroit with means (SDs) of 35.50 (3.26), 19.60 (5.06), 21.50 (1.68), and a rate of 2032.00 (116.00 per 100,000), respectively. In Seattle the mean rates (SDs) of diabetes, physical inactivity smoking, and unemployment were the lowest at 6.62 (0.34), 13.40 (0.66), 10.80 (57.20), and 4.98 (1.00), respectively.

**Figure 1:**
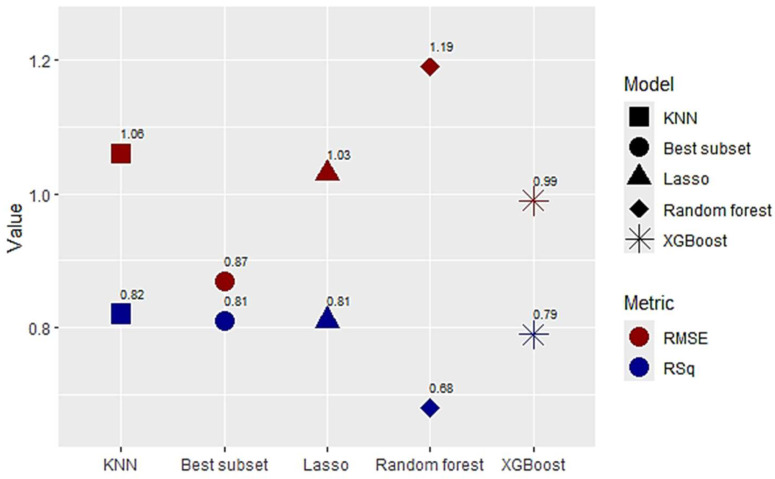
Performance metrics of five machine learning approaches for the prediction of the prevalence of low birth weight in the big cities of the USA. KNN—K-nearest neighbors, XGBoost—Extreme Gradient Boosting, RMSE—residual root mean squared error, RSq—R-squared.

Figure 1 displays the performance metrics (RSq, RMSE) of the five trained ML approaches on the test set. The models showed excellent performance with RSq results of 0.82, 0.81, 0.81, and 0.79, and RMSE results of 1.06, 0.87, 1.03, 0.99 for KNN, Best subset, Lasso, and XGBoost, respectively. The Best subset selection approach had a high RSq, but the lowest RMSE, with a subset of only four predictors. The Random forest model demonstrated a slightly worse performance with an RSq of 0.68 and an RMSE of 1.19. Table 2 shows tuning parameters selected by a grid search of the training set for each model.

### Importance of the Predictors

**Figure 2:**
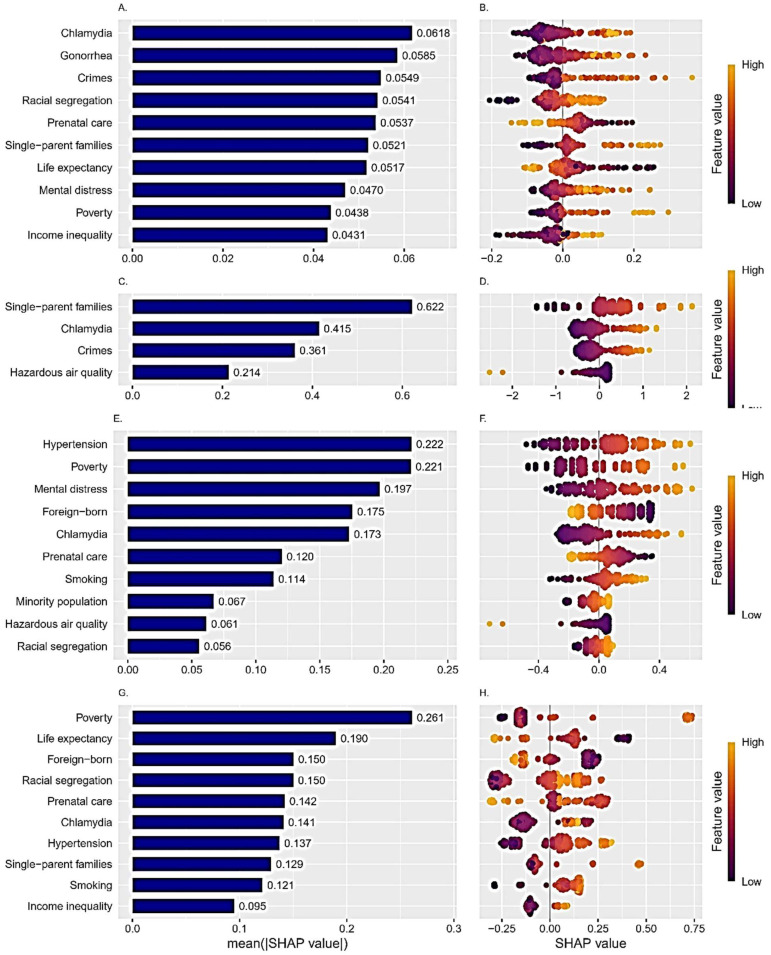
Most influential predictors from the models with the best predictive performance. Average variable importance (SHAP value) (left column) and individual SHAP values (right column) from the K-nearest neighbors ((**A**,**B**), respectively), best subset ((**C**,**D**), respectively), Lasso ((**E**,**F**), respectively), and Extreme gradient boosting ((**G**,**H**), respectively) models. In the right column the dot’s position on the X axis shows the positive or negative impact of each individual measurement on the prediction and the color corresponds to value.

Figure 2 presents an analysis of the importance of the variables in the best performing models using the model-agnostic approach, i.e., KNN, Best subset, Lasso and XGBoost. For each model (except for the Best subset in which the best performance was observed with four predictors), the top ten most influential predictors are shown. The left column of plots shows the absolute mean SHAP values of the predictors. The higher the value, the more significant the influence of this predictor in the model. The right column represents a set of beeswarm plots, where each dot corresponds to an individual subject in the study. The color represents the size of the feature value (purple color—low values, and yellow—higher values of the feature). The dots’ positions on the X axis show the positive or negative impact of each individual measurement on the prediction. The combination of the two columns facilitates comprehension of the strength and direction of the impact. The rate of chlamydia infection was an influential predictor of LBW rate in all four models. High rates of infection were associated with a higher prevalence of LBW. Influential predictors that appear in three of four models are racial segregation, single-parent families, poverty (high values were linked to higher prevalence of LBW), and prenatal care (low values were linked to higher prevalence of LBW). Several predictors (violent crime rate, life expectancy, mental distress, income inequality, hazardous air quality, prevalence of hypertension, percent of foreign-born citizens, and smoking) were influential in two models, with positive association for all, except life expectancy, where low values were associated with a higher prevalence of LBW.

## 4. Discussion

LBW is a public health problem of global concern. A multitude of health-related, social, environmental, and demographic factors are associated with it. In the present study, the performance of five different ML algorithms was evaluated, at the population level, in predicting the prevalence of LBW. Four of the five evaluated models had excellent predicting performance: KNN, best subset selection, and Lasso had an RSq of above 0.80, and XGboost 0.79. One of the simplest approaches, best subset selection, which had the lowest RMSE (0.87), predicted LBW with only four variables: single-parent families, rate of chlamydia infection, violent crimes rate, and hazardous air quality. All these variables were influential in the other models of the study as well. Single-parent family was reported as a risk-factor for LBW in two studies [21,22], though living without a partner had no effect on LBW in a population-level study from Spain [23]. A large body of evidence exists on the association of infection with chlamydia and multiple adverse pregnancy outcomes including LBW [24,25]. Several studies had previously demonstrated the negative impact of maternal exposure to neighborhood crime on infant weight [26,27,28]. Common environmental exposures, exacerbated by climate change, demonstrated significant associations with serious adverse pregnancy outcomes across the US and in other countries [11,29,30]. It should be noted that almost all the factors with the largest impact on models’ predictive performances were influential predictors in more than one model. The association of these factors with LBW is supported by strong scientific evidence as well [16,29,31,32,33,34]. The important predictors found in the present study belonged to different domains that were related to health/morbidity, and environmental, demographic, and socio-economic factors, highlighting the complexity of the problem of LBW and the need for a multidisciplinary approach to it.

Identification of the most influential predictors at the population level can inform healthcare authorities on more effective ways to tackle LBW. Proposed interventions include screening for chlamydia infections, which was found to be an influential predictor in four models, or increasing the rates of prenatal care visits, e.g., by cash-transfer programs. Screening and early treatment of mental distress and hypertension and the promotion of smoking cessation among women of childbearing age could also be helpful. At the municipality level, interventions should focus on decreasing racial segregation and support for single-parent families.

Large for gestational age births are also associated with short- and long-term complications, so future studies should address the predictive capacity of the ML approach to this problem [35].

Limitations and strengths. The main limitation of the present study was assessing LBW as a single entity, not dividing it into term and preterm LBW births. These two obstetric conditions have many common causes, including maternal malnutrition, poverty, black race, narrow child spacing, multiple gestations, low maternal education, poor antenatal care, substance abuse, smoking, emotional and physical stress, and illnesses. Some of the causes, however, are unique to preterm LBW (induction or cesarean section for maternal complications such as pre-eclampsia, some infectious and inflammatory processes, including chorioamnionitis, bacterial vaginosis, bacteriuria) [7]. Unfortunately, the BCHI data platform only includes data on preterm births since 2016, hence this factor could not be included. Another reservation is the questionable external validity of our findings to other settings, such as rural settings or small cities. Large urban areas tend to have better health outcomes with urban populations often enjoying higher access to healthcare, social services, and economic opportunities. However, urban areas can also face challenges like increased risk of infectious diseases and pollution-related health issues. Thus, the impact of the examined factors on LBW can be different in different settings. On the other hand, the assessment of the predictive ability of ML approaches on the prevalence of LBW at the population level in large cities was the goal of the present study.

Among the strengths of the study are the multitude of ML algorithms and predictors from different domains that were assessed, including predictors that were not included in previous studies with an ML approach.

## 5. Conclusions

KNN, Best subset, Lasso, and XGBoost ML algorithms showed excellent performance for the prediction of LBW rates in the big cities. Identification of the influential predictors, especially those that are significant in several models, could help local and state authorities and health policy decision makers to tackle this important health problem more effectively.

## Figures and Tables

**Table 1:** Descriptive statistics of the study variables.

Variables	Mean (SD)	City with Highest Value Mean (SD)	City with Lowest Value Mean (SD)
Health/morbidity-related:			
Low birth weight, percent	9.11 (1.89)	Detroit 14.2 (0.83)	Seattle 6.51 (0.27)
Prenatal care, percent	73.38 (9.21)	Oakland 89.30 (2.05)	Houston 58.4 (1.89)
Obesity, percent	30.60 (6.51)	Detroit 35.5 (1.53)	San Francisco 18.1 (0.93)
Diabetes, percent	10.81 (2.64)	Detroit 18.00 (0.33)	Seattle 6.62 (0.34)
Hypertension, percent	30.14 (5.49)	Detroit 46.30 (0.79)	Austin 22.8 (0.51)
Physical inactivity, percent	25.23 (5.60)	Detroit 36.60 (1.22)	Seattle 13.40 (0.66)
Flu vaccinations, percent	47.0 (6.10)	Minneapolis 56.20 (3.56)	El Paso 32.70 (1.93)
New chlamydia cases, rate per 100,000	737.98 (261.75)	Baltimore 1299.00 (121.00)	San Jose 367.00 (58.50)
New gonorrhea cases, rate per 100,000	264.92 (139.89)	Baltimore 528.00 (146.00)	El Paso 87.50 (23.00)
Binge drinking, percent	17.65 (2.88)	Washington 22.4 (131.00)	Memphis 11.9 (79.60)
Smoking, percent	18.09 (4.64)	Detroit 29.00 (68.80)	Seattle 10.80 (57.20)
Mental distress, percent	14.11 (2.56)	Detroit 18.6 (1.52)	San Francisco 10.80 (1.45)
Life expectancy at birth, years	77.80 (3.05)	San Jose 82.90 (0.55)	Detroit 72.20 (1.51)
Climate/built environment related:			
City parks system, index (0–100)	55.53 (15.70)	Minneapolis 83.70 (92.44)	Indianapolis 30.20 (1.75)
Poor air quality, number of days	45.36 (20.50)	Long Beach 90.30 (0.80) Los Angeles 90.30 (1.11)	Portland 13.80 (0.83)
Hazardous air quality, number of days	5.33 (8.06)	Long Beach 30.00 (5.38) Los Angeles 30.00 (5.38)	Minneapolis 0.34 (0.34)
Social and economic:			
Community social vulnerability to climate disasters, percent	39.54 (14.18)	Detroit 69.9 (12.20)	Seattle 10.90 (2.47)
Housing lead risk, percent	29.26 (19.77)	Cleveland 63.8 (2.72)	Las Vegas 1.63 (0.12)
Uninsured, percent	11.65 (5.26)	Dallas 24.40 (0.70)	Boston 3.65 (0.30)
College graduates, percent	36.17 (11.77)	Seattle 62.40 (4.07)	Detroit (4.8 (1.87)
Poverty level, percent	18.89 (5.66)	Detroit 35.50 (3.26)	San Jose 9.63 (1.54))
Unemployment, percent	8.05 (3.38)	Detroit 19.60 (5.06)	Seattle 4.98 (1.00)
Per capita household income, dollars	35,546.27 (12,981.37)	San Francisco 66,946.00 (16,553.00)	Detroit 18,543.00 (3607.00)
Income inequality, index (0–100)	0.49 (0.03)	New York City 0.547 (0.00	Columbus 0.45 (0.00)
Owners occupied housing, percent	48.15 (7.32)	Louisville 60.5 (0.84)	New York City 32.70 (0.24)
Excessive housing cost, percent	30.04 (4.03)	Los Angeles 42.4 (0.67)	Oklahoma City 22.80 (0.14)
Single parent families, percent	12.89 (3.69)	Detroit 21.50 (1.68)	San Francisco 5.09 (0.34)
Teen births, percent	29.59 (15.57)	Houston 53.1 (16.3)	Seattle 6.15 (3.19)
Violent crimes, rate per 100,000	890.89 (449.98)	Detroit 2032.00 (116.00)	El Paso 370.00 (55.30)
Demographic:			
Population density, per square mile	6325.13 (5365.88)	New York City 28,250 (668.00)	Oklahoma City 1052 (66.40)
Children under 5 years, percent	22.16 (3.46)	Fort Worth 27.90 (1.03)	San Francisco 13.50 (0.07)
Minority population, percent	60.24 (14.51)	Detroit 90.40 (0.83)	Portland 29.80 (1.70)
Foreign-born, percent	18.51 (9.37)	San Jose 39.80 (1.13)	Cleveland 5.34 (0.55)
White-Black Racial segregation, Index 0–100	47.58 (9.30)	Memphis 61.50 (1.28)	Portland 25.70 (2.20)

**Table 2:** Tuning parameters selected for the models.

Model	Tuning Parameter	Value
K-nearest neighbors	K (number of neighbors)	45
Best subset selection	Number of predictors	4
Lasso	Λ (penalty term)	0.125
Random forest	Number of predictors at each split time.	5
XGBoost	Maximum depth of a tree	4
	Minimum number of observations in a split	27
	Gamma (minimum loss reduction in the split)	0
	Eta (learning rate)	0.05
	Maximum number of iterations	1400
	Fraction of predictors in each tree	0.3
	Fraction of observations in each tree	0.5

## Data Availability

The data that support the findings of this study are available in Big Cities Health Inventory Data Platform at Bigcitieshealthdata.org, reference number [12].

## Supplementary Materials

The following supporting information can be downloaded at: https://www.mdpi.com/article/10.3390/ijerph22060934/s1, Table S1: Source and descriptions of predictors for low birth weight; Table S2: Details of missing values for predictors in the original data
