# Supplementary material for: Predicting Low Birth Weight in Big Cities in the United States Using a Machine Learning Approach
Source: Int J Environ Res Public Health. 2025 Jun 13;22(6):934. doi: 10.3390/ijerph22060934 (PMC12192627; doi:10.3390/ijerph22060934)
Supplement: Supplementary file 1 [file ijerph-22-00934-s001.zip › ijerph-3632757-supplementary.pdf]

Supplementary Table S1. Source and Descriptions of predictors for low birth weight

Predictor	***Predictor description***	***Data source***	***Missing datapoints (N, %)***
Health/morbidity related:			
Prenatal care	Percent of live births where prenatal care began by the third month of pregnancy	National Vital Statistics System	18 (3.96)
Obesity	Percent of adults aged 18+ who are obese	Centers for Disease Control and Prevention's Population Level Analysis and Community Estimates	None
Diabetes	Percent of adults aged 20+ with diabetes	Centers for Disease Control and Prevention's Population Level Analysis and Community Estimates	None
Hypertension	Percent of adults aged 18+ with high blood pressure	Centers for Disease Control and Prevention's Population Level Analysis and Community Estimates	None
Physical inactivity	Percent of adults aged 18+ who didn't participate in any leisure-time physical activity	Centers for Disease Control and Prevention's Population Level Analysis and Community Estimates	None

Flu vaccinations	Percent of fee-for-service Medicare enrollees who had flu vaccination	Centers for Medicare and Medicaid Services of the U.S. Department of Health and Human Services.	None
New chlamydia cases	Crude rate per 100,000 population aged 13+ years	National Center for HIV/AIDS, Viral Hepatitis, STD, and TB Prevention Atlas Plus of the Centers for Disease Control and Prevention	3 (0.66)
New gonorrhea cases	Crude rate per 100,000 population aged 13+ years	National Center for HIV/AIDS, Viral Hepatitis, STD, and TB Prevention Atlas Plus of the Centers for Disease Control and Prevention	3 (0.66)
Binge drinking	Percent of women who drank more than 4 and men who drank more than 5 drinks in 30 days, among adults aged 18+ years	Centers for Disease Control and Prevention's Population Level Analysis and Community Estimates	None
Smoking	Percent of respondents aged 18+ years who smoked ≥100 cigarettes in their lifetime and currently smoke	Centers for Disease Control and Prevention's Population Level Analysis and Community Estimates	None
Mental distress	Percent of adults aged 18+ years who report their mental health as	Centers for Disease Control and Prevention's	None

	being “not good” for 14 or more days during the past 30 days	Population Level Analysis and Community Estimates	
Life expectancy	Life expectancy at birth	National Vital Statistics System of the National Center for Health Statistics	35 (7.69)
Climate/built environment related:			
City parks system	Index scaled from 0 – 100, based on analysis of acreage, investment, amenities, and access of park system	ParkScore database of the Trust for Public Land	None
Poor air quality	Number of days in which the Air Quality index was >50	Environmental Protection Agency	1 (0.22)
Hazardous air quality	Number of days when the Air Quality index was >100	Environmental Protection Agency	1 (0.22)
Community social vulnerability to climate disasters	Percent with a high index of need for support during a hazardous event, based on socioeconomic, household, racial/ethnic, and transportation characteristics	Agency for Toxic Substance and Disease Registry’s Geospatial Research, Analysis, & Services Program	None
Housing lead risk	Percent of housing units built before 1950	U.S. Census Bureau’s American Community Survey 5-year estimates	None
Social and economic:			
Uninsured	Percent of the population, of all ages,	U.S. Census Bureau’s American Community	105 (23.08)

	who didn't have private or public insurance	Survey 5-year estimates	
College graduates	Percent of people aged 25+ who reported having attained a bachelor's degree or higher	U.S. Census Bureau's American Community Survey 5-year estimates	None
Poverty level	Percent of the population, of all ages, who reported their family income for the past 12 months to be below the federal poverty level	U.S. Census Bureau's American Community Survey 5-year estimates	None
Unemployment	Percent of people in the civilian workforce aged 16+ who reported being currently unemployed	U.S. Census Bureau's American Community Survey 5-year estimates	None
Per-capita household income	Per-capita income for the total population	U.S. Census Bureau's American Community Survey 5-year estimates	None
Income inequality	GINI index (0-100)-the extent to which the distribution of income among individuals deviates from a perfectly equal distribution	U.S. Census Bureau's American Community Survey 5-year estimates	None
Owners occupied housing	Percent of occupied housing units occupied by the owner	U.S. Census Bureau's American Community Survey 5-year estimates	None
Excessive housing cost	Percent of renters who reported the rent, or owner-occupied housing	U.S. Census Bureau's American Community	

	units who reported the mortgage to be >35% of the household income	Survey 5-year estimates	
Single parent families	Percent of households with a child below 18 with female only or male only householder	U.S. Census Bureau's American Community Survey 5-year estimates	None
Teen births	Percent of live births to mothers aged 15 to 19	National Vital Statistics System of the National Center for Health Statistics	None
Violent crimes	Crude rate of murder, aggravated assault, robbery, and rape per 100,000 citizens	Uniform Crime Reporting program of the Federal Bureau of Investigation	14 (3.08)
Demographic:			
Population density	Total population of the city divided by the land area of the city.	U.S. Census Bureau's American Community Survey 5-year estimates; gazetteer files published by the U.S. Census Bureau	None
Children	Percent of the population aged less than 5 years	U.S. Census Bureau's American Community Survey 5-year estimates	None
Minority population	Percent of people who didn't report being non-Hispanic white	U.S. Census Bureau's American Community Survey 5-year estimates	None
Percent of foreign-born	Percent of people who reported being born in a foreign country	U.S. Census Bureau's American Community Survey 5-year estimates	None

White-Black Racial segregation	Index of dissimilarity (0-100) which reflects the evenness with which Black and White residents are distributed across the neighborhoods	U.S. Census Bureau's American Community Survey 5-year estimates; Missouri Census Data Center of the University of Missouri	None

Supplementary Table S2. Details of missing values for predictors in the original data

Predictor	***Missing values, Cities (years)***
Health/morbidity-related	
Prenatal care	Boston (2010,2011), Charlotte (2010, 2011), Milwaukee (2010, 2011), Minneapolis (2010, 2011), Phoenix (2010, 2011, 2012, 2013, 2014), Tucson (2010, 2011, 2012, 2013, 2014)
Obesity	None
Diabetes	None
Hypertension	None
Physical inactivity	None
Flu vaccinations	None
New chlamydia cases	Washington (2014, 2015, 2016)
New gonorrhea cases	Washington (2014, 2015, 2016)
Binge drinking	None
Smoking	None
Mental distress	None
Climate/built environment related:	Austin (2010), Baltimore (2010), Boston (2010), Charlotte (2010) , Chicago (2010), Cleveland (2010), Columbus (2010), Dallas (2010), Denver (2010), Detroit (2010), El Paso (2010), Fort Worth (2010), Houston (2010) , Indianapolis(2010), Kansas city (2010), Las Vegas (2010), Long Beach (2010), Los Angeles (2010), Louisville (2010), Memphis (2010), Milwaukee (2010), Minneapolis (2010), New York City (2010), Oakland (2010), Oklahoma City (2010), Philadelphia (2010), Phoenix (2010), Portland (2010), San Antonio (2010), San Diego(2010), San Francisco (2010), San Jose (2010), Seattle (2010), Tucson (2010), Washington (2010)
Climate/built environment related:	
City parks system	None
Poor air quality	Louisville (2022)

Hazardous air quality	Louisville (2022)
Community social vulnerability to climate disasters	None
Housing lead risk	None
Social and economic	
Uninsured	Austin (2010, 2011, 2012), Baltimore (2010, 2011, 2012), Boston (2010, 2011, 2012), Charlotte (2010, 2011, 2012) , Chicago (2010, 2011, 2012), Cleveland (2010, 2011, 2012), Columbus (2010, 2011, 2012), Dallas (2010, 2011, 2012), Denver (2010, 2011, 2012), Detroit (2010, 2011, 2012), El Paso (2010, 2011, 2012), Fort Worth (2010, 2011, 2012), Houston (2010, 2011, 2012), Indianapolis(2010, 2011, 2012), Kansas city (2010, 2011, 2012), Las Vegas (2010, 2011, 2012), Long Beach (2010, 2011, 2012), Los Angeles (2010, 2011, 2012), Louisville (2010, 2011, 2012), Memphis" (2010, 2011, 2012), Milwaukee (2010, 2011, 2012), Minneapolis (2010, 2011, 2012), New York City (2010, 2011, 2012), Oakland (2010, 2011, 2012), Oklahoma City (2010, 2011, 2012), Philadelphia (2010, 2011, 2012), Phoenix (2010, 2011, 2012), Portland (2010, 2011, 2012), San Antonio (2010, 2011, 2012), San Diego(2010, 2011, 2012), San Francisco (2010, 2011, 2012), San Jose (2010, 2011, 2012), Seattle (2010, 2011, 2012), Tucson (2010, 2011, 2012),<br>Washington (2010, 2011, 2012)
College graduates	None
Poverty level	None
Unemployment	None
Per-capita household income	None
Income inequality	None
Owners occupied housing	None

Excessive housing cost	Austin (2010, 2011, 2012), Baltimore (2010, 2011, 2012), Boston (2010, 2011, 2012), Charlotte (2010, 2011, 2012) , Chicago (2010, 2011, 2012), Cleveland (2010, 2011, 2012), Columbus (2010, 2011, 2012), Dallas (2010, 2011, 2012), Denver (2010, 2011, 2012), Detroit (2010, 2011, 2012), El Paso (2010, 2011, 2012), Fort Worth (2010, 2011, 2012), Houston (2010, 2011, 2012), Indianapolis(2010, 2011, 2012), Kansas city (2010, 2011, 2012), Las Vegas (2010, 2011, 2012), Long Beach (2010, 2011, 2012), Los Angeles (2010, 2011, 2012), Louisville (2010, 2011, 2012), Memphis" (2010, 2011, 2012), Milwaukee (2010, 2011, 2012), Minneapolis (2010, 2011, 2012), New York City (2010, 2011, 2012), Oakland (2010, 2011, 2012), Oklahoma City (2010, 2011, 2012), Philadelphia (2010, 2011, 2012), Phoenix (2010, 2011, 2012), Portland (2010, 2011, 2012), San Antonio (2010, 2011, 2012), San Diego(2010, 2011, 2012), San Francisco (2010, 2011, 2012), San Jose (2010, 2011, 2012), Seattle (2010, 2011, 2012), Tucson (2010, 2011, 2012), Washington (2010, 2011, 2012)
Single parent families	None
Teen births	None
Violent crimes	Baltimore (2021), Chicago (2021), Long Beach (2021), Los Angeles (2021), New York City (2021), Oakland (2021), Philadelphia (2021), Phoenix (2021), San Francisco (2021), San Jose (2021), Tucson (2014,2021, 2022), Washington (2021)
Demographic	
Population density	None
Children	None
Minority population	None
Percent of foreign-born	None
White-Black Racial segregation	None
